# A Genetic Folding Strategy Based Support Vector Machine to Optimize Lung Cancer Classification

**DOI:** 10.3389/frai.2022.826374

**Published:** 2022-06-30

**Authors:** Mohammad A. Mezher, Almothana Altamimi, Ruhaifa Altamimi

**Affiliations:** ^1^Computer Science Department, Fahd Bin Sultan University, Tabuk, Saudi Arabia; ^2^Department of Clinical Medicine and Surgery, Università Degli Studi di Napoli Federico II, Naples, Italy; ^3^Department of Business and Data Analytics, University of Huddersfield, Huddersfield, United Kingdom

**Keywords:** genetic folding algorithm, evolutionary algorithms, lung cancer, classification, genetic programming, support vector machine

## Abstract

Cancer is defined as an abnormal growth of human cells classified into benign and malignant. The site makes further classification of cancers of initiation and genomic underpinnings. Lung cancer displays extreme heterogeneity, making genomic classification vital for future targeted therapies. Especially considering lung cancers account for 1.76 million deaths worldwide annually. However, tumors do not always correlate to cancer as they can be benign, severely dysplastic (pre-cancerous), or malignant (cancerous). Lung cancer presents with ambiguous symptoms, thus is difficult to diagnose and is detected later compared to other cancers. Diagnosis relies heavily on radiology and invasive procedures. Different models developed employing Artificial Intelligence (AI), and Machine Learning (ML) have been used to classify various cancers. In this study, the authors propose a Genetic Folding Strategy (GFS) based model to predict lung cancer from a lung cancer dataset. We developed and implemented GF to improve Support Vector Machines (SVM) classification kernel functions and used it to classify lung cancer. We developed and implemented GF to improve SVM classification kernel functions and used it to classify lung cancer. Classification performance evaluations and comparisons between the authors' GFS model and three SVM kernels, linear, polynomial and radial basis function, were conducted thoroughly on real lung cancer datasets. While using GFS in classifying lung cancer, the authors obtained an accuracy of 96.2%. This is the highest current accuracy compared to other kernels.

## Introduction

Cancer is characterized by the uncontrolled growth of various human cells that can either be malignant or benign (Adjiri, 2016). Malignant cancers are the most aggressive type as they can invade surrounding tissue and reach different organs of the body. At the same time, benign cancers stay limited to the original tissue (Cooper, 2000). Some cancers can develop rapidly with no apparent symptoms, hence why earlier detection is associated with a better prognosis (Bhattacharjee and Majumder, 2019).

Lung cancer accounts for 2 million new cases and 1.76 million deaths worldwide per year (Sung et al., 2021). The incidence rate has been declining since the introduction of anti-smoking measures. However, lung cancer still amounted to 18% of deaths caused by cancers in 2020 (Sung et al., 2021). Lung cancer's etiology is highly complex due to its heterogeneity and is still not realized. It is understood that constant exposure to carcinogens leads to pre-cancerous cells (dysplasia) in the lung epithelium (Siddiqui and Siddiqui, 2021).

The dysplastic cells can form cancerous cells if the exposure causes a mutation in the genes that regulate the cell cycle, thus promoting carcinogenesis. The most common gene mutations are in KRAS (29%), EGFR (17%), BRAF (15%), MET (~4%), and other non-identified genes account for 32% (Thai et al., 2021). Lung cancer confers clinicopathological heterogeneity hence the difficulty in precise classification. However, most lung cancers are classified broadly as non-small cell lung cancer (NSCLC), which constitutes 85% of cases, and small cell lung cancer (SCLC), which constitutes 15 percent of cases. NSCLC is categorized into five types; squamous cell carcinoma, adenocarcinoma, adenosquamous carcinoma, large cell carcinoma, and carcinoid tumors (Siddiqui and Siddiqui, 2021; Thai et al., 2021).

Lung cancer is difficult to diagnose early since it presents no unusual symptoms during its development. At the time of diagnosis, the majority of patients have an advanced stage of the disease, with the most common symptoms being cough (8–75%), hemoptysis (6–35%), dyspnea (3–60%), and chest pain (20–49%); Hammerschmidt and Wirtz, 2009). Therefore, lung cancer diagnosis depends heavily on radiology, including PET or CT scans. Invasive surgical procedures are used to diagnose and treat lung cancer, including bronchoscopy, mediastinoscopy, thoracoscopy, and others (Siddiqui and Siddiqui, 2021). Therapies vary for the treatment of lung cancer depending on the key oncotic driver genes. For example, Osimertinib is used for EGFR-driven lung cancers, whereas Dabrafenib with Trametinib is used for BRAF-driven lung cancers (Thai et al., 2021).

AI with its child ML is at the pinnacle of revolutionizing healthcare with the help of technology. Many ML models have allowed for early-stage diagnosis and prediction of numerous cancers to improve prognosis (Tataru et al., 2021). Of these models, Support Vector Machine (SVM) and Artificial Neural Networks (ANN) have been used in the classification of lung cancer using X-Ray images with an accuracy of 98.08% (Nanglia et al., 2021). Improvised Crow Search Algorithm (ICSA) and Improvised Gray Wolf Algorithm (IGWA) were also used in automated lung disease detection with accuracies of 99 and 99.4%, respectively. Although out of the 111 features, only 45 were used in the ICSA model and 53 in the IGWA model (Gupta et al., 2019).

In 2019, (Lakshmanaprabu et al., 2019) used an optimal deep learning classification method to categorize lung cancer. They received a 94.56% accuracy score; conclusively, they found that an automatic lung cancer classification approach reduced the manual labeling time, ultimately avoiding human error. The outcomes were effective for accuracy, sensitivity and specificity, achieving 94.56%, 96.2 and 94.2%, respectively. Radhika et al. (2019) conducted a comparative study to showcase efficiencies in Logistic Regression, SVM, decision tree and Naïve Bayes algorithm models, achieving 66.7, 90, 87.87, and 99.2%, respectively. Their research highlighted those doctors can limit the amount of testing conducted on a patient in order to classify them with cancer or not. These algorithms will reduce the number of unnecessary check-ups a patient need. The performance of the SVM yielded the best result and can be taken as a means for lung cancer detection.

The confusion matrix used by Günaydin et al. (2019) was the most commonly used method of evaluating the performance of models from datasets that generated a confusion matrix for each technique and evaluated accuracy accordingly. Günaydin et al. (2019) used different ML methods to detect lung cancer from chest radiographs. They found that reducing dimension would cause feature loss in chest radiographs; hence they used PCA to reduce dimension at a ratio of 1:8. This increased accuracy in KNN and SVM. Conclusively, Decision Tree had the highest result performance measurements in comparison to other neural nets. Using the WEKA tool, alongside several classification techniques (Murty and Babu, 2017), the Naive Bayesian algorithm performs better than other classification algorithms. However, other data mining techniques such as Times Series/Clustering could have further enhanced the prediction system.

Linning et al. (2019) gathered 278 patients with pathologically confirmed lung cancer, including 181 non-small cell lung cancer (NSCLC) and 97 small lung cancer (SCLC) patients. Overall, 1,695 quantitative radiomic features were considered for lung cancer in each patient. A result of 74.1% was achieved for the AUC model, SCLC vs. NSCLC. This showed that phenotypic differences exist among different types of lung cancer subtypes on non-enhanced computed tomography images. Singh and Gupta (2019) used a dataset of 15,750 clinical images, 6,910 benign, and 8,840 malignant lung cancer-related images to test and train classifiers. They found that the accuracy of Multi-Layer Perceptron (MLP) was 88.55% compared to other classifies (support vector machine classifier, decision tree classifier, multinomial naive Bayes classifier, stochastic gradient descent classifier, and random forest classifier).

There are many features included in this dataset, and this article will use an array of those symptoms and features such as age, smoking, yellow fingers, anxiety, fatigue, allergy, alcohol, and chronic disease, among many others. This article aims to use SVM to improve the classification accuracy of GF in classifying lung cancer into malignant and benign.

## Support Vector Machine (SVM)

The Support vector machines are essential to the genetic folding process (Mezher and Abbod, 2011). Support Vector Machines (SVM) were the first introduced by Vapnik (1998). SVM is a supervised ML classification approach commonly used in cancer diagnosis and prognosis. SVM works by identifying key samples (support vectors) from all classes and separating them by developing a function that separates them as widely as feasible using these support vectors. As a result, it is possible to say that SVM is used to create a mapping between an input vector and a high dimensionality space to find the most suited hyperplane that splits the data set into classes (Hammerschmidt and Wirtz, 2009). This linear classifier maximizes the distance between the decision hyperplane and the closest data point, known as the marginal distance, by locating the best-matched hyperplane (Nangliaa et al., 2018).

An SVM classifier divides a collection of training vectors into two pairs of data points (*x*_1_, *y*_1_), (*x*_2_, *y*_2_), …(*x*_*m*_, *y*_*m*_) where $x_{i}\in R^{d}\;$signifies vectors in a d-dimensional feature space and *y*_*i*_∈{−1, +1} is a class label. The SVM model is created by translating the input vectors onto a new higher dimensional feature space designated as *F*:*R*^*d*^→*H*^*d*^′, where *d* < *d*′. A kernel function *K*(*x*_*i*_, *x*_*j*_), which is the dot product of input vectors *x*_*i*_ and *x*_*i*_, constructs an optimum separating hyperplane in the new feature space φ(*x*_*i*_), φ(*x*_*j*_) (Radhika et al., 2019).

The Kernel Trick (Murty and Babu, 2017) is a mathematical technique that trains a classifier in a higher-dimensional space. It works by calculating the distance (the scalar products) of the data for the enlarged feature representation without ever doing the expansion. The preset kernels may be divided into three well-known kernels. The most frequent are linear, polynomial, and Radial Basis Functions (RBF) (Kourou et al., 2015).

This paper compares various kernels to classify the lung cancer dataset. The biggest challenge that must be overcome while training an SVM using predefined kernels is determining the appropriate values for the two hyperparameters: C and gamma. The hyperparameter C is shared by all SVM kernels, and balances the misclassification training example against the hyperplane surface. A low C value smoothes the hyperplane surface, while a high C value attempts to identify all training samples accurately. Gamma defines the magnitude of the effect of a single training example, the greater the gamma value, the closer additional instances must be to be influenced. Relevant researches demonstrate that there is no formal method for selecting kernel functions. The kernel functions used are determined by the data and the particular domain issue. The GF algorithm will be used to build new kernels without the requirement for such hyperparameters to be addressed.

## The Proposed Genetic Folding Strategy Algorithm (GFS)

An Evolution Strategy (ES) is a computer science optimization approach based on an evolutionary lifecycle. It falls within the broad category of evolutionary computation or artificial evolution approaches. As search operators, evolution techniques use natural problem-dependent representations, namely mutation and selection. Evolution strategies have been successfully applied in various application areas, e.g., Gorunescu and Belciug (2014).

**Figure 1:**
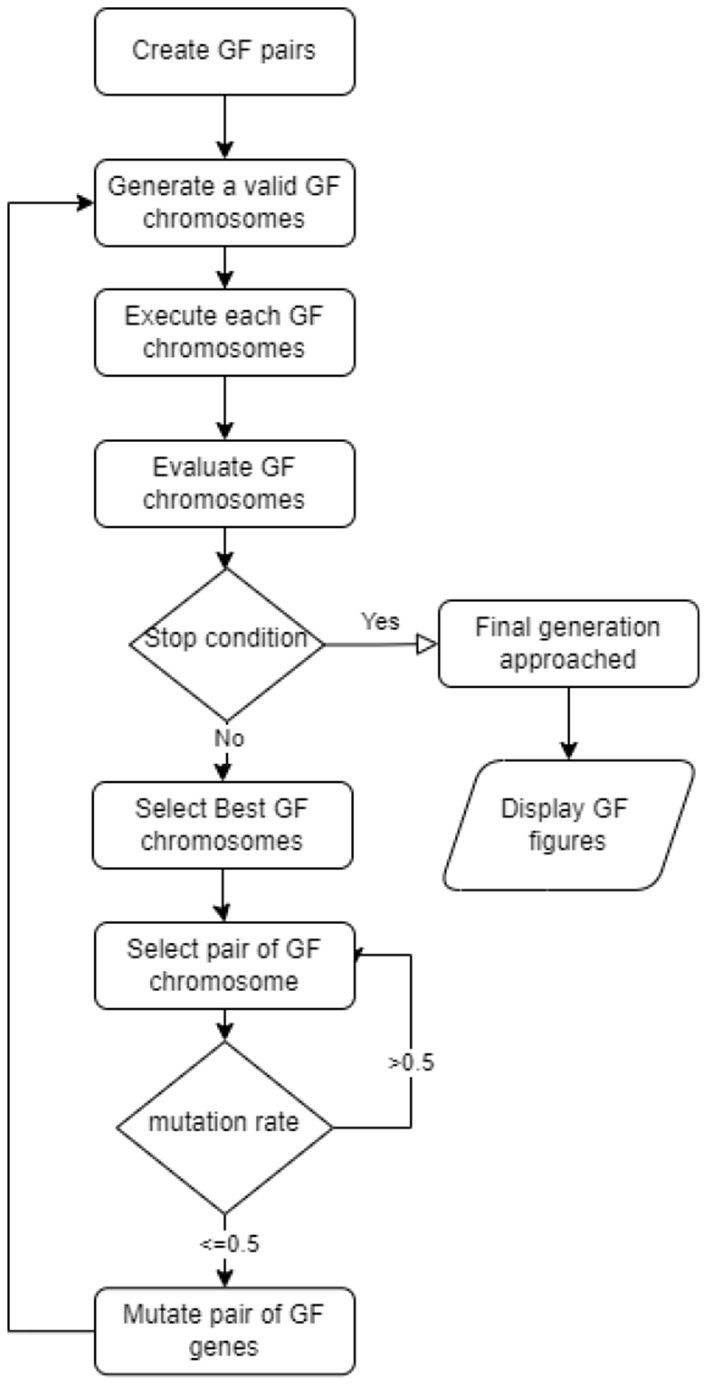
GF life cycle.

GF denotes the new variation of the genetic algorithm (Mezher and Abbod, 2011). GF solved a variety of real-world classification and regression problems. GFS uses the evolutionary concept of linear pairings of terminals. Figure 1 depicts the full GF life cycle (Mezher, 2022) of the seven steps proposed in the new GFS version were:

1. Select pairings of the non-terminal and terminal list, respectively;
  a. operators = {+_v, +_s, -_v, -_s, ^*^_s}, and
  b. operands = {x, y}.

2. Create a valid set of pairings that results in a valid GFS chromosome format, e.g.:
  a. {2.3, 4.5, 3, 6.7, 5, 6, 7}
  b. {^*^_s, -_s, x, +_v, x, y, x}

3. A fitness function that works with the produced terminals.
4. Select the best GFS chromosomes
5. Mutate the GFS chromosomes
6. Stopping criterion (number of generations approached)
7. Repeat steps 2–5 until step 6 is satisfied.

The table of parameters used in each step of the life cycle is shown in Table 1. There are some columns where the values are objects, and we transformed these values into numerical values. The dataset has no null values to be treated in which we apply the standard scaler library found in Python. The Sparse data types are specifically avoided in the preprocessing computations. Therefore, the preprocessing was designed by organizing a sequential and recurring manner of processing data into scaler modules.

**Table 1:** A list of parameters and values used in the experiments.

**Parameters**	**Values**
Operators	{+_v, +_s, - v, - s, *_s}
Operands	{x, y}
Fitness function	Apply the produced equation to the datapoints
Selection function	Roulette wheel selection
Mutation function	Mutate at less than or equals to 0.5 ratios for each operator and operand
Stopping criterion	300 generations
No. of generations	20
No. of populations	50
Mutation rate	0.5
Scaler	Sklearn.preprocessing.StandardScaler()
K-Folds	5-folds

## Experiments Setup

All experiments in this research were carried out using a laptop computer equipped with a Windows 10 Pro and Intel(R) Core (TM) i7-8550U CPU @ 1.80 GHz, 1992 MHz, 4 Core(s), 8 Logical Processors, 16.0 GB of DDR5 RAM, and a GPU NVIDIA GeForce MX150 GPU Memory 11.9 GB. The Python programming language implements itself using Visual Studio code. The project included around 25 .py script files programmed to allow the GF to forecast the optimal lung categorization. Most of these scripts start by reading data from the Binary folder, then modeling it with various parameters, and finally producing an individual kernel that represents the output of the binary.py script.

We excluded all patients with null values from the dataset in the preprocessing stage, totalling 33 patients. There were 39 benign patients and 270 malignant ones. To that aim, the lung cancer dataset, with a ratio of 0.159, is deemed unbalanced. This implies that the outcome is far from one, and we must adjust for this during the preprocessing step. Table 2 shows a sample of the lung cancer dataset prior to being in the experiments. Note that the data presented in the table are for illustration purposes only; it is worth noting that some data entries may be changed to respect the confidentiality of scaling patients' samples.

**Table 2:** Lung cancer dataset sample.

**Out**	**F_1**	**F_2**	**F_3**	**F_4**	**F_5**	**F_6**	**F_7**	**F_8**	**F_9**	**F_10**	**F_11**	**F_12**	**F_13**	**F_14**	**F_15**
YES	M	69	1	2	2	1	1	2	1	2	2	2	2	2	2
YES	M	74	2	1	1	1	2	2	2	1	1	1	2	2	2
NO	F	59	1	1	1	2	1	2	1	2	1	2	2	1	2
NO	M	63	2	2	2	1	1	1	1	1	2	1	1	2	2
NO	F	63	1	2	1	1	1	1	1	2	1	2	2	1	1

In Table 3, we illustrated a dataset sample after applying the scaled function (Table 1). Table 4 describes the characteristics given in Table 2 used in our tests. The table shows the value of each attribute and its average value.

**Table 3:** Lung cancer scaled dataset sample.

**Out**	**F_1**	**F_2**	**F_3**	**F_4**	**F_5**	**F_6**	**F_7**	**F_8**	**F_9**	**F_10**	**F_11**	**F_12**	**F_13**	**F_14**	**F_15**
YES	0.953	0.772	1.135	0.869	1.003	1.003	1.010	0.697	1.120	0.892	0.892	0.852	0.749	1.064	0.892
YES	0.953	1.382	0.881	1.150	0.997	1.003	0.990	0.697	0.892	1.120	1.120	1.173	0.749	1.064	0.892
NO	1.050	0.448	1.135	1.150	0.997	0.997	1.010	0.697	1.120	0.892	1.120	0.852	0.749	0.940	0.892
NO	0.953	0.040	0.881	0.869	1.003	1.003	1.010	1.435	1.120	1.120	0.892	1.173	1.336	1.064	0.892
NO	1.050	0.040	1.135	0.869	0.997	1.003	1.010	1.435	1.120	0.892	1.120	0.852	0.749	0.940	1.120

**Table 4:** A list of features found in the lung cancer dataset (Bhat, 2021).

**Shortcut**	**Feature**	**Value**	**Mean**
F_1	Gender	M(male), F(female)	-
F_2	Age	Age of the patient	62.6
F_3	Smoking	YES=2, NO=1	1.5
F_4	Yellow fingers	YES=2, NO=1	1.5
F_5	Anxiety	YES=2, NO=1	1.5
F_6	Peer_pressure	YES=2, NO=1	1.5
F_7	Chronic Disease	YES=2, NO=1	1.5
F_8	Fatigue	YES=2, NO=1	1.8
F_9	Allergy	YES=2, NO=1	1.5
F_10	Wheezing	YES=2, NO=1	1.5
F_11	Alcohol	YES=2, NO=1	1.5
F_12	Coughing	YES=2, NO=1	1.6
F_13	Shortness of breath	YES=2, NO=1	1.6
F_14	Swallowing difficulty	YES=2, NO=1	1.5
F_15	Chest pain	YES=2, NO=1	1.5
Out	Lung cancer	YES, NO	-

Each row gives a single example of the feature details. However, the average age in the data samples is 62.6 years, as shown in Table 4.

**Figure 2:**
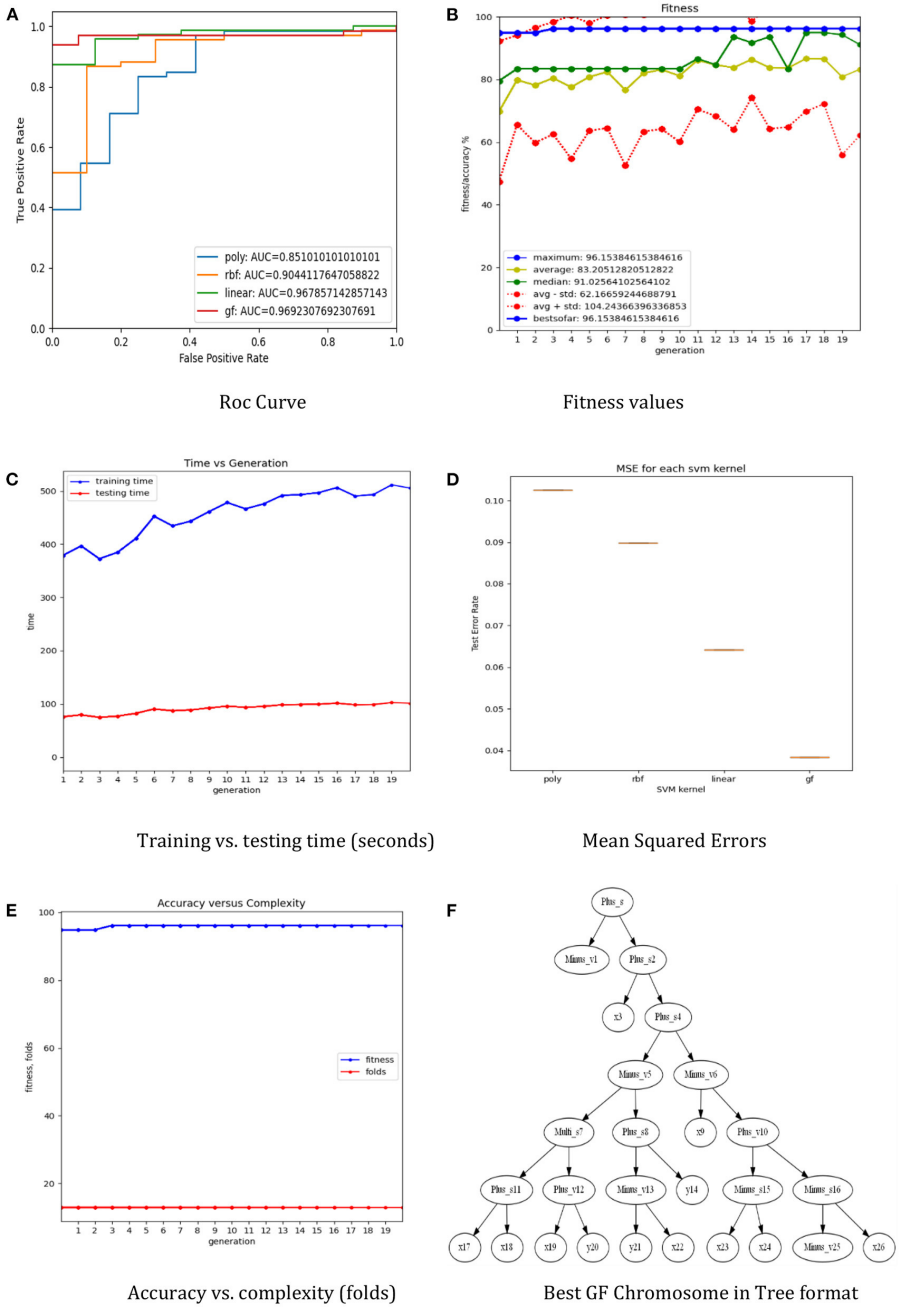
The results of the GF toolbox for the lung cancer dataset. **(A)** Roc curve. **(B)** Fitness values. **(C)** Training vs. testing time (seconds). **(D)** Mean squared errors. **(E)** Accuracy vs. complexity (folds). **(F)** Best GF chromosome in tree format.

The dataset was separated using a 5-fold cross-validation technique. The training set is used to train the three preset SVM kernel functions as well as a generic optimal GFS kernel, and the test set is used to evaluate performance using accuracy (Figure 2B), ROC (Figure 2A), mean squared errors (Figure 2D), process time (Figure 2C), and the complexity of the generic GFS kernel (Figure 2E). The best GFS chromosome found is shown in Figure 2F. Furthermore, the evaluation metrics are generated using the GFS life cycle in each generation, with appropriate hyperparameters configured to provide the best GFS kernel, as shown in Table 1. From the results, GFS can provide a 6% gain in accuracy over the SVM RBF kernel with an improvement in mean squared error. It can also notice a 5-fold increase in complexity over the training phase at the expense of an additional 10 generations and 50% less test time.

Table 5 illustrates that the suggested GFS algorithm outperformed the other algorithms evaluated in the Kaggle contests (Bhat, 2021). The performance of the RF is higher than conventional kernels, linear, RBF, and polynomial in terms of accuracy score, with 95.8%, compared to 93.6, 91.0, and 89.7%, respectively. Furthermore, there is very little performance difference between GFS and RF regarding accuracy. Table 5 shows the performance comparison of all ML models for the same scaled lung dataset. The proposed GFS model achieved an average accuracy score of 96% (Figure 2B), ranked in the top 3 across all Kaggle contests. In addition, the AUC value was 97% (Figure 2A), which also ranks in the top 3 across all Kaggle contests. Also, the mean square error was at the minimum of errors compared to the rest (Figure 2D). Thus, the proposed technique gives an alternate model to the present ML methods. Based on the findings, it is apparent that the proposed GFS model can significantly improve upon current approaches when used in lung cancer applications. The random forest and quadratic discriminant analysis performed the best subsequent algorithms, 95.8 and 96.1%, respectively (Table 5). The model for quadratic discriminant analysis was constructed in R and carried out using the EDA approach. Meanwhile, the random forest model used the hyperparameter approach to increase accuracy. However, when the random over sampler approach was conducted, the KNeighbors classifier achieved an accuracy of 93.5%. The standard deviations of the models show that the diversity is around ±2 and was greater than most of the other modes, which means that the standard deviations of the error distributions are influenced by the mean in the SVM and the proposed model.

**Table 5:** List of parameters and values used in the experiments.

**Model**	**Accuracy (%)**	**Standard deviations**
**GFS**	**96.2**	**2.03**
Random forest (Santos, 2021)	95.8	0.05
SVM (linear)	93.6	2.38
SVM (RBF)	91.0	2.76
SVM (polynomial)	89.7	2.17
Logistic regression (Santos, 2021)	84.0	0.0
Gaussian NB (Santos, 2021)	84.0	0.0
Gradient boosting (Santos, 2021)	84.0	0.0
KNeighbors (Santos, 2021)	76.5	0.0
AdaBoost (Santos, 2021)	76.5	0.1
Linear regression (Bhatt, 2021)	64.0	0.0
KNeighbors classifier (Bhatt, 2021)	93.5	0.0
Quadratic discriminant analysis (Wu, 2021)	96.1	0.0

*Best are in bold*.

## Conclusion

This study showed that using the GF algorithm in lung cancer classification garnered a higher accuracy level (96.2%) than Random Forest, SVM, Logistic Regression, Linear regression Gaussian NB, Gradient Boosting, KNeighbours, QDA and AdaBoost. These results advocate the use of AI and ML in healthcare. In the future, this may be used to enable clinicians to better stratify patients with lung cancers to provide better-targeted therapies. This was the first study to demonstrate that the GFS approach was effective in lung cancer prognosis, and it was more accurate than compared classifiers. Cross dataset validation was used to measure reliability. The GFS approach using the mutation operator alone beats other kernels in accuracy and area under the curve according to the experimental data. The GFS model provides a definitive answer to lung cancer prediction concerns with an accuracy of 96.2%.

## Data Availability Statement

The datasets presented in this study can be found in online repositories. The names of the repository/repositories and accession number(s) can be found in the article/supplementary material.

## Author Contributions

All authors listed have made a substantial, direct, and intellectual contribution to the work and approved it for publication.

## Conflict of Interest

The authors declare that the research was conducted in the absence of any commercial or financial relationships that could be construed as a potential conflict of interest.

## Publisher's Note

All claims expressed in this article are solely those of the authors and do not necessarily represent those of their affiliated organizations, or those of the publisher, the editors and the reviewers. Any product that may be evaluated in this article, or claim that may be made by its manufacturer, is not guaranteed or endorsed by the publisher.
